# Sex differences in clinical profile across Lewy body disease pathology stages

**DOI:** 10.1016/j.nbas.2026.100175

**Published:** 2026-11-02

**Authors:** Ece Bayram, Danelle J. Carter, Irene Litvan

**Affiliations:** a Department of Neurology, University of Colorado Anschutz, 12469 E 19^th^ Ave, Aurora, CO 80045, USA; b Department of Neurosciences, University of California San Diego, 9500 Gilman Dr, La Jolla, CA 92093, USA

**Keywords:** Lewy body, Alzheimer's, Dementia, Pathology, Clinicopathological correlation, Sex

## Abstract

**Background:**

Sex impacts clinical profile for people with limbic, neocortical LB pathology, and AD pathology contributes to heterogeneity. Amygdala, olfactory, and brainstem LB can also underlie LB disease and sex differences for these pathology stages are unclear.

**Methods:**

Data were obtained from National Alzheimer's Coordinating Center for 198 females and 314 males with LB pathology and available data on AD pathology staging. Main and interaction effects of sex and LB pathology group for clinical diagnosis, cognitive, behavioral, and motor outcomes were assessed with logistic and linear regression models, with and without AD pathology staging as a covariate. Sex differences within individual LB pathology groups were also assessed.

**Results:**

Females were less likely to have spouses/partners as informants, had less advanced LB pathology stage, and more advanced ADNC stage. Females were less likely to have RBD and a clinical diagnosis of LB dementia, including after adjustment for AD pathology stage. Advancing LB pathology was associated with a greater reduction in the likelihood of clinical AD diagnosis and a greater increase in the likelihood of auditory hallucinations in males than females, after adjustment for AD pathology stage. Females with neocortical LB pathology were less likely than males to have RBD.

**Conclusions:**

Lower likelihood of RBD in females was particularly evident at the neocortical LB pathology stage. Clinical correlates of LB pathology may differ by sex, and these sex differences may not be fully explained by AD co-pathology. Considering sex and co-pathology is important for both diagnostic and therapeutic approaches to LB disease.

## 1 Introduction

Lewy body (LB) pathology is the neuropathological hallmark of LB diseases, including Parkinson's disease (PD), Parkinson's disease dementia (PDD), and dementia with Lewy bodies (DLB) [1]. PD is clinically characterized by progressive levodopa-responsive parkinsonism accompanied by a range of motor and non-motor symptoms impacting daily life [2]. LB dementia, including DLB and PDD, is characterized by non-amnestic dementia, cognitive fluctuations, well-formed visual hallucinations, rapid eye movement sleep behavior disorder (RBD), and parkinsonism [1]. Currently, clinical diagnostic accuracy for LB diseases during life is limited, and neuropathological assessment remains the gold standard for diagnosis [1]. This diagnostic challenge is particularly pronounced in females, who have higher rates of delayed or misdiagnosis than males [3].

In addition to sex, co-occurring Alzheimer's disease (AD) pathology impacts the clinical profile in LB diseases. AD pathology can occur in over half of individuals with LB pathology and contributes to clinical heterogeneity [4]. The prevalence, severity and clinical correlates of both LB and AD pathologies differ by sex [3], [5], [6]. Female sex is associated with a higher likelihood of AD pathology and a higher likelihood that AD pathology is accompanied by a late-onset amnestic dementia profile [5]. In our previous work including individuals with limbic or neocortical LB pathology, females were less likely than males to exhibit a typical LB disease clinical profile or cognitive decline despite similar LB and AD pathology stages [3]. Whether these sex differences extend across the full spectrum of LB pathology remains unclear. Although limbic and neocortical LB pathology are more strongly associated with a LB disease clinical profile, olfactory-only, amygdala and brainstem-predominant LB pathology may also occur in individuals with LB disease [7]. Thus, in this study, we examined individuals with amygdala, olfactory, brainstem, limbic, or neocortical LB pathology in the National Alzheimer's Coordinating Center (NACC). Our primary goal was to determine whether clinical profiles differed according to sex and LB pathology group with a focus on sex and LB pathology interactions. Because AD co-pathology has sex-specific clinical correlates [3] and may modify the clinical profile in LB disease [4], we performed these analyses adjusting for National Institute on Aging-Alzheimer's Association AD Neuropathologic Change (ADNC).

## 2 Materials & methods

### 2.1 Participants

We obtained data from the NACC Neuropathology Data Set and Uniform Data Set for visits conducted between September 2005 and August 2025 [8], [9], [10]. The study was approved by the institutional review board at each participating data collection site and conducted in accordance with the 1964 Declaration of Helsinki. Written informed consent was obtained from participants prior to inclusion.

We included participants with 1) confirmed LB pathology irrespective of clinical diagnosis, 2) available ADNC staging [11] or Braak neurofibrillary tangle (NFT) staging when ADNC was unavailable, 3) no other neurodegenerative pathologies considered to contribute substantially to cognitive decline. Excluded neuropathologies included frontotemporal lobar degeneration, other tauopathies, TDP-43 pathology, pigment-spheroid degeneration, multiple system atrophy, prion disease, trinucleotide disease, cortical development malformation, metabolic/storage disorder, white matter disease (leukodystrophy, multiple sclerosis, other demyelinating disease), acute or chronic contusion/traumatic brain injury, primary/metastatic neoplasm, infectious process, herniation, and Down syndrome.

These criteria resulted with 198 females and 314 males from 10 AD Research Centers. Participants were classified according to LB pathology staging as 1) amygdala (51 females, 65 males); 2) olfactory (21 females, 21 males); 3) brainstem (20 females, 40 males); 4) limbic (35 females, 43 males); 5) neocortical (71 females, 145 males).

### 2.2 Clinical measures

To characterize the clinical profile closest to neuropathological assessment, we analyzed data from the last clinical visit before death. Outcomes included clinician-reported diagnoses; age at cognitive decline onset; and cognitive, behavioral, and motor symptoms documented at the last visit. Clinician assessments were based on examination and interviews with participants and informants. Dementia severity was assessed with CDR® Dementia Staging Instrument-Sum of Boxes (CDR-SOB). Behavioral symptom severity was assessed with Neuropsychiatric Inventory Questionnaire total score (NPI-Q).

### 2.3 Statistics

Analyses were performed using R version 4.4.1. False discovery rate (FDR) correction was used to account for multiple comparisons. FDR-adjusted *q* values <0.05 were considered statistically significant. Demographic and neuropathological characteristics were compared across LB pathology groups and between sexes using Chi square, t, Mann Whitney U, and Kruskal Wallis tests, as appropriate.

The primary variables of interest were sex, LB pathology group, and interaction between sex and LB pathology group. Linear and logistic regression models were used to examine the association of these primary variables of interest with clinical outcomes. Age at last clinical visit, and informant relationship to participant (spouse/partner or other) were included as covariates in all models. Years of education was included as an additional covariate for cognitive outcomes including age at cognitive decline onset, cognitive symptoms, and CDR-SOB. To assess whether AD co-pathology contributed to these associations, the models were repeated with ADNC included as an additional covariate.

In secondary analyses, we examined sex differences within each LB pathology group in linear and logistic regression models with clinical outcomes and covariates as described above. As in the main analyses, models were constructed both with and without ADNC as an additional covariate. Due to the smaller sample sizes within each LB pathology group, both unadjusted *p* values and FDR-adjusted *q* values are reported.

## 3 Results

### 3.1 Sex and LB pathology group comparisons for demographics and pathology staging (Table 1)

The majority of the cohort identified as non-Hispanic (95.5%) and White (92.2%) and had informants at last visit (98.4%).

**Table 1:** Demographics, clinical diagnosis at last visit and neuropathology across the Lewy body (LB) pathology groups and sexes.

	Amygdala LB (*n* = 116)		Olfactory LB (*n* = 42)		Brainstem LB (*n* = 60)		Limbic LB (*n* = 78)		Neocortical LB (*n* = 216)	
	Females (*n* = 51)	Males (*n* = 65)	Females (*n* = 21)	Males (n = 21)	Females (*n* = 20)	Males (*n* = 40)	Females (*n* = 35)	Males (*n* = 43)	Females (*n* = 71)	Males (*n* = 145)
Age at last visit	71.9 (11.0)	68.2 (10.7)	75.3 (13.6)	70.7 (12.0)	85.1 (11.5)	81.9 (10.2)	72.0 (13.5)	77.0 (9.5)	77.0 (9.9)*	73.5 (8.5)*
Age at death	75.3 (10.4)	71.8 (10.1)	76.7 (13.1)	72.4 (11.7)	85.9 (11.3)	83.7 (10.1)	75.8 (12.7)	79.2 (9.5)	79.6 (10.3)*	75.7 (8.2)*
Years of education	15.6 (3.1)	16.4 (2.8)	14.1 (2.3)*	16.3 (2.7)*	14.5 (3.3)	16.3 (2.9)	15.3 (2.9)*	17.3 (2.4)*	15.2 (2.5)	16.0 (3.1)
Hispanic ethnicity	5 (9.8%)	2 (3.1%)	0	1 (4.8%)	3 (15.0%)	0	2 (5.7%)	0	3 (4.2%)	4 (2.8%)
Race										
-American Indian or Alaska Native	1 (2.0%)	0	0	0	0	0	0	0	0	0
-Asian	0	2 (3.1%)	0	0	1 (5.0%)	2 (5.0%)	0	0	0	0
-Black	3 (5.9%)	0	3 (14.3%)	1 (4.8%)	0	1 (2.5%)	5 (14.3%)	2 (4.7%)	9 (12.7%)	6 (4.1%)
-White	44 (86.3%)	62 (95.4%)	18 (85.7%)	20 (95.2%)	19 (95.0%)	37 (92.5%)	30 (85.7%)	41 (95.3%)	62 (87.3%)	139 (95.9%)
Has informant at last visit	50 (98.0%)	63 (96.9%	21 (100%)	21 (100%)	18 (90.0%)	40 (100%)	35 (100%)	43 (100%)	69 (97.2%)*	144 (99.3%)*
Informant's relationship to participant, spouse/partner	29 (58.0%)*	55 (87.3%)*	14 (66.7%)	17 (81.0%)	6 (33.3%)	28 (70.0%)	19 (54.3%)*	37 (86.0%)*	34 (49.3%)*	122 (84.7%)*
ADNC stage							*	*	*	*
-None	1 (2.0%)	0	0	0	2 (10.0%)	3 (7.5%)	2 (5.7%)	4 (9.3%)	0	8 (5.5%)
-Low	0	0	1 (4.8%)	0	4 (20.0%)	9 (22.5%)	1 (2.9%)	9 (20.9%)	4 (5.6%)	23 (15.9%)
-Intermediate	4 (7.8%)	4 (6.2%)	1 (4.8%)	0	7 (35.0%)	13 (32.5%)	4 (11.4%)	8 (18.6%)	21 (29.6%)	43 (29.7%)
-High	46 (90.2%)	61 (93.8%)	19 (90.5%)	21 (100.0%)	7 (35.0%)	15 (37.5%)	28 (80.0%)	22 (51.2%)	46 (64.8%)	71 (49.0%)

Variables are reported as mean (standard deviation) or number (percentage), as appropriate. Statistically significant sex differences within each LB pathology group are marked with * for false discovery rate corrected *q* < 0.05. LB: Lewy body, ADNC: National Institute on Aging- Alzheimer's Association Alzheimer's disease neuropathologic change [11]

#### 3.1.1 Sex comparison

A higher percentage of females reported Hispanic ethnicity and Black race than males (*q* = 0.042, *q* = 0.009). Females were less likely to have spouses/partners as informants, had fewer years of education, less advanced LB pathology stage, and more advanced ADNC stage (*q* < 0.042 for all). Age at last visit and death did not differ between females and males (*q* = 0.11, *q* = 0.066).

#### 3.1.2 LB pathology group comparison

Age at last visit and death, race, and ADNC stage differed across LB pathology groups (*q* < 0.018 for all). Participants in the brainstem LB group were oldest and had the lowest ADNC stage. ADNC stage was highest for the amygdala and olfactory LB pathology groups. Years of education, ethnicity, and informant relationship were similar across LB pathology groups (*q* > 0.36 for all).

#### 3.1.3 Sex comparison within LB pathology groups

In the amygdala, limbic, and neocortical LB groups, females were less likely to have their spouse/partner as their informant (*q* < 0.034 for all). In the olfactory and limbic LB groups, females had fewer years of education (*q* = 0.034, *q* = 0.017, respectively). In the neocortical LB group, females were older at the last visit and death (*q* = 0.042, *q* = 0.035). In the limbic and neocortical LB groups, females had more advanced ADNC stage (*q* = 0.037, *q* = 0.033). There were no other sex differences in demographic characteristics or ADNC stage within LB pathology groups (*q* > 0.066 for all).

### 3.2 Sex and LB pathology group effect on clinical profile at last visit

Main and interaction effects of sex and LB pathology stage in models with and without ADNC adjustment are shown in Table 2.

**Table 2:** Clinical features affected by sex and Lewy body (LB) pathology stage in models with and without National Institute on Aging-Alzheimer's Association Alzheimer's disease neuropathologic change (ADNC) as a covariate.

	**Main effect of sex**		**Main effect of LB pathology stage**		**Interaction of sex and LB pathology**	
	**Without ADNC**	**With ADNC**	**Without ADNC**	**With ADNC**	**Without ADNC**	**With ADNC**
**Cognitive outcomes**						
CDR-SOB	B (SE) = 1.52 (0.55), *p =* .006*	B (SE) = 1.05 (0.54), *p =* .052	B (SE) = −0.041 (0.16), *p =* .79	B (SE) = 0.23 (0.16), *p =* .14	B (SE) = 0.28 (0.32), *p =* .38	B (SE) = 0.064 (0.31), *p =* .83
Cognitive fluctuations	OR (95% CI) = 0.64 (0.39, 1.05), *p =* .080	OR (95% CI) = 0.76 (0.45, 1.27), *p =* .29	OR (95% CI) = 1.46 (1.25, 1.71), *p <* .001*	OR (95% CI) = 1.35 (1.15, 1.61), *p <* .001*	OR (95% CI) = 0.94 (0.68, 1.32), *p =* .72	OR (95% CI) = 1.01 (0.72, 1.43), *p =* .95
**Behavioral outcomes**						
Total NPI-Q	B (SE) = 0.52 (0.58), *p =* .37	B (SE) = 0.52 (0.58), *p =* .38	B (SE) = 0.48 (0.16), *p =* .003*	B (SE) = 0.52 (0.17), *p =* .002*	B (SE) = −0.54 (0.33), *p =* .11	B (SE) = −0.57 (0.34), *p =* .090
Delusions	OR (95% CI) = 0.93 (0.56, 1.52), *p =* .77	OR (95% CI) = 0.93 (0.56, 1.53), *p =* .79	OR (95% CI) = 1.29 (1.11, 1.52), *p =* .001*	OR (95% CI) = 1.31 (1.12, 1.54), *p =* .001*	OR (95% CI) = 0.88 (0.64, 1.21), *p =* .42	OR (95% CI) = 0.87 (0.63, 1.20), *p =* .38
Visual hallucinations	OR (95% CI) = 0.79 (0.50, 1.24), *p =* .31	OR (95% CI) = 0.88 (0.56, 1.40), *p =* .60	OR (95% CI) = 1.57 (1.35, 1.84), *p <* .001*	OR (95% CI) = 1.52 (1.30, 1.79), *p <* .001*	OR (95% CI) = 0.76 (0.55, 1.04), *p =* .088	OR (95% CI) = 0.78 (0.56, 1.07), *p =* .12
Auditory hallucinations	OR (95% CI) = 2.54 (1.23, 5.32), *p =* .012	OR (95% CI) = 2.43 (1.17, 5.13), *p =* .018	OR (95% CI) = 1.07 (0.87, 1.34), *p =* .52	OR (95% CI) = 1.11 (0.90, 1.40), *p =* .34	OR (95% CI) = 0.40 (0.17, 0.73), *p =* .010	OR (95% CI) = 0.40 (0.17, 0.72), *p =* .008*
Rapid eye movement sleep behavior disorder	OR (95% CI) = 0.30 (0.15, 0.55), *p <* .001*	OR (95% CI) = 0.35 (0.17, 0.66), *p =* .002*	OR (95% CI) = 1.59 (1.34, 1.92), *p <* .001*	OR (95% CI) = 1.43 (1.18, 1.75), *p <* .001*	OR (95% CI) = 0.59 (0.39, 0.89), *p =* .011	OR (95% CI) = 0.61 (0.39, 0.94), *p =* .024

**Motor outcomes**						
Falls	OR (95% CI) = 0.76 (0.48, 1.20), *p =* .25	OR (95% CI) = 0.87 (0.54, 1.39), *p =* .56	OR (95% CI) = 1.29 (1.12, 1.49), *p <* .001*	OR (95% CI) = 1.22 (1.05, 1.42), *p =* .009*	OR (95% CI) = 0.69 (0.51, 0.92), *p =* .012	OR (95% CI) = 0.71 (0.52, 0.96), *p =* .028
Gait changes	OR (95% CI) = 0.74 (0.50, 1.10), *p =* .13	OR (95% CI) = 0.85 (0.57, 1.26), *p =* .42	OR (95% CI) = 1.42 (1.27, 1.61), *p <* .001*	OR (95% CI) = 1.35 (1.20, 1.53), *p <* .001*	OR (95% CI) = 0.84 (0.66, 1.07), *p =* .16	OR (95% CI) = 0.88 (0.69, 1.12), *p =* .30
Bradykinesia	OR (95% CI) = 0.66 (0.44, 0.98), *p =* .040	OR (95% CI) = 0.75 (0.50, 1.13), *p =* .17	OR (95% CI) = 1.50 (1.33, 1.69), *p <* .001*	OR (95% CI) = 1.42 (1.25, 1.61), *p <* .001*	OR (95% CI) = 0.88 (0.69, 1.13), *p =* .30	OR (95% CI) = 0.92 (0.72, 1.18), *p =* .50
Tremors	OR (95% CI) = 0.65 (0.41, 1.03), *p =* .071	OR (95% CI) = 0.72 (0.45, 1.15), *p =* .17	OR (95% CI) = 1.45 (1.26, 1.68), *p <* .001*	OR (95% CI) = 1.40 (1.21, 1.64), *p <* .001*	OR (95% CI) = 0.87 (0.64, 1.18), *p =* .37	OR (95% CI) = 0.89 (0.65, 1.21), *p =* .44

**Clinical diagnosis**						
Lewy body dementia	OR (95% CI) = 0.46 (0.29, 0.71), *p <* .001*	OR (95% CI) = 0.54 (0.33, 0.87), *p =* .012*	OR (95% CI) = 2.22 (1.86, 2.71), *p <* .001*	OR (95% CI) = 2.17 (1.78, 2.71), *p <* .001*	OR (95% CI) = 0.87 (0.59, 1.32), *p =* .49	OR (95% CI) = 0.92 (0.60, 1.45), *p =* .69
-Dementia with Lewy bodies	OR (95% CI) = 0.50 (0.30, 0.80), *p =* .005*	OR (95% CI) = 0.59 (0.35, 0.98), *p =* .045	OR (95% CI) = 2.31 (1.88, 2.94), *p <* .001*	OR (95% CI) = 2.28 (1.82, 2.96), *p <* .001*	OR (95% CI) = 0.79 (0.50, 1.28), *p =* .31	OR (95% CI) = 0.80 (0.49, 1.34), *p =* .37
Alzheimer's disease	OR (95% CI) = 1.38 (0.90, 2.12), *p =* .14	OR (95% CI) = 0.95 (0.58, 1.57), *p =* .85	OR (95% CI) = 0.56 (0.47, 0.65), *p <* .001*	OR (95% CI) = 0.59 (0.49, 0.71), *p <* .001*	OR (95% CI) = 1.68 (1.21, 2.36), *p =* .002*	OR (95% CI) = 1.80 (1.21, 2.76), *p =* .005*

For main effect of sex and sex X LB pathology interaction, male sex was the reference group. Findings are reported as odds ratio (95% confidence interval) or B (standard error), as appropriate. Models with cognitive outcomes included age at last visit, informant relationship to participant (spouse/partner or not) and years of education as covariates. Models with clinical diagnosis, behavioral and motor outcomes included age at last visit and informant relationship to participant (spouse/partner or not) as covariates. “With ADNC” column depicts models with ADNC staging as an additional covariate. Rows are shown when at least one model has *p <* .05. Effects with false discovery rate corrected *q* < 0.05 are marked with *. CDR-SOB: CDR® Dementia Staging Instrument-Sum of Boxes, NPI-Q: Neuropsychiatric Inventory-Questionnaire, OR: odds ratio, CI: confidence interval, SE: standard error.

#### 3.2.1 Main effect of sex

Female sex was associated with a lower likelihood of RBD, clinical diagnosis of LB dementia and DLB, and higher CDR-SOB scores in models without ADNC adjustment (*q* < 0.031 for all). After adjustment for ADNC, females continued to have a lower likelihood of RBD and clinical diagnosis of LB dementia (*q* < 0.042 for both).

#### 3.2.2 Main effect of LB pathology stage

More advanced LB pathology stage was associated with a greater likelihood of cognitive fluctuations, delusions, visual hallucinations, RBD, falls, gait changes, bradykinesia, tremors, clinical diagnosis of LB diseases, higher NPI-Q scores, and a lower likelihood of clinical AD diagnosis in models without ADNC adjustment (*q* ≤ 0.033). After adjustment for ADNC, associations with LB dementia related clinical features, clinical diagnosis of LB dementia including DLB, and clinical AD diagnosis remained significant (*q* < 0.033 for all).

#### 3.2.3 Interaction effect of sex and LB pathology stage

The interaction between sex and LB pathology stage was significant for only AD clinical diagnosis (*q* = 0.017). More advanced LB pathology stage was associated with a lower likelihood of clinical AD diagnosis in males relative to females. After adjustment for ADNC, this association remained significant (*q* = 0.032) and there was also a significant interaction for the likelihood of auditory hallucinations (*q* = 0.047) (Fig. 1). The association between advancing LB pathology stage and greater odds of auditory hallucinations was more pronounced in males.

**Fig. 1:**
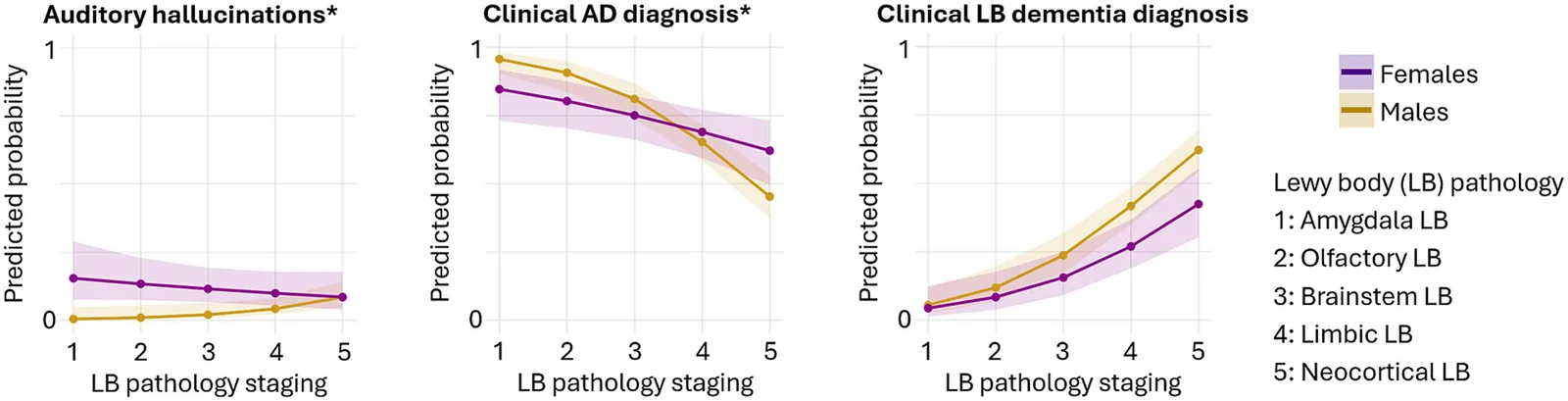
Predicted probabilities for auditory hallucinations, clinical Alzheimer's disease (AD) and Lewy body (LB) dementia diagnoses based on sex and Lewy body pathology interaction models. Predicted values and 95% confidence intervals are shown by sex across LB pathology stages. Models were adjusted for age at last visit, informant relationship to participant (spouse/partner or not), and National Institute on Aging-Alzheimer's Association AD Neuropathologic Change (ADNC) stage. Significant sex and Lewy body pathology interactions are marked with * for false discovery rate-corrected *q* < 0.05.

### 3.3 Sex differences for clinical profile within each LB pathology group

Clinical variables at last visit for sexes within each pathology group are shown in Table 3. Clinical outcomes differing by sex within individual LB pathology groups with and without ADNC adjustment are shown in Table 4.

**Table 3:** Clinical features at last visit and for females and males across Lewy body (LB) pathology groups.

	Amygdala LB (*n* = 116)		Olfactory LB (*n* = 42)		Brainstem LB (*n* = 60)		Limbic LB (*n* = 78)		Neocortical LB (*n* = 216)	
	Female (*n* = 51)	Male (*n* = 65)	Female (*n* = 21)	Male (n = 21)	Female (*n* = 20)	Male (*n* = 40)	Female (*n* = 35)	Male (*n* = 43)	Female (*n* = 71)	Male (*n* = 145)
**Cognitive outcomes**^**a**^										
Dementia	47 (92.2%)	61 (93.8%)	20 (95.2%)	21 (100.0%)	15 (75.0%)	31 (77.5%)	32 (91.4%)	35 (81.4%)	67 (94.4%)	131 (90.3%)
CDR-SOB	10.7 (5.57)	9.57 (5.04)	13.3 (5.65)	13.9 (4.00)	11.2 (7.24)	8.85 (6.46)	11.8 (5.87)	8.94 (5.85)	11.8 (5.01)	9.92 (5.37)
Age at cognitive decline onset	63.9 (10.3)	61.4 (9.88)	65.7 (12.2)	61.7 (10.6)	76.3 (11.4)	73.8 (10.4)	61.7 (10.0)	68.2 (10.6)	70.0 (10.1)	66.5 (8.51)
Memory change	50 (98.0%)	62 (95.4%)	20 (95.2%)	21 (100.0%)	17 (85.0%)	36 (90.0%)	31 (88.6%)	39 (90.7%)	69 (97.2%)	136 (93.8%)
Orientation change	33 (97.1%)	38 (86.4%)	0	0	3 (42.9%)	16 (88.9%)	13 (81.2%)	16 (69.6%)	21 (77.8%)	52 (82.5%)
Judgment change	48 (96.0%)	61 (93.8%)	19 (90.5%)	21 (100.0%)	15 (75.0%)	32 (80.0%)	32 (91.4%)	39 (90.7%)	69 (97.2%)	134 (92.4%)
Language change	39 (78.0%)	49 (75.4%)	15 (71.4%)	20 (95.2%)	13 (65.0%)	23 (57.5%)	27 (77.1%)	28 (65.1%)	57 (80.3%)	101 (70.1%)
Visuospatial change	34 (73.9%)	48 (77.4%)	18 (85.7%)	18 (85.7%)	12 (63.2%)	25 (62.5%)	26 (74.3%)	31 (72.1%)	54 (78.3%)	112 (77.8%)
Attention change	33 (71.7%)	45 (73.8%)	15 (71.4%)	18 (85.7%)	10 (50.0%)	24 (60.0%)	22 (62.9%)	32 (78.0%)	56 (78.9%)	115 (79.9%)
Cognitive fluctuation	4 (9.3%)	6 (10.5%)	3 (21.4%)	5 (33.3%)	4 (20.0%)	8 (23.5%)	5 (15.2%)	11 (26.2%)	21 (33.9%)	50 (42.0%)

**Behavioral outcomes**^**a**^										
Total NPI-Q	6.90 (5.59)	5.88 (4.69)	6.81 (5.50)	7.81 (6.22)	6.75 (7.40)	4.47 (4.11)	7.37 (6.68)	5.98 (7.08)	7.13 (6.14)	8.41 (6.45)
Visual hallucination	6 (12.5%)	4 (6.3%)	5 (25.0%)	4 (19.0%)	3 (15.8%)	11 (27.5%)	5 (14.3%)	10 (23.8%)	25 (35.7%)	64 (44.4%)
Auditory hallucination	6 (12.5%)	1 (1.6%)	4 (20.0%)	1 (4.8%)	3 (15.8%)	0	3 (8.6%)	2 (4.9%)	6 (8.8%)	13 (9.2%)
Delusion	3 (6.2%)	6 (9.5%)	7 (35.0%)	4 (19.0%)	2 (10.5%)	5 (12.8%)	8 (22.9%)	6 (14.6%)	15 (21.7%)	38 (26.6%)
Disinhibition	9 (18.8%)	9 (14.3%)	5 (25.0%)	4 (19.0%)	4 (20.0%)	4 (10.0%)	11 (31.4%)	7 (16.7%)	12 (17.1%)	25 (17.5%)
Irritability	19 (38.0%)	21 (32.8%)	7 (35.0%)	10 (47.6%)	8 (40.0%)	7 (17.5%)	12 (34.3%)	13 (31.0%)	21 (29.6%)	47 (33.1%)
Agitation	14 (28.0%)	13 (20.0%)	7 (35.0%)	6 (28.6%)	6 (30.0%)	4 (10.0%)	11 (31.4%)	10 (23.3%)	22 (31.0%)	36 (25.4%)
Personality change	7 (14.0%)	9 (13.8%)	2 (10.5%)	6 (28.6%)	4 (20.0%)	2 (5.0%)	6 (17.6%)	9 (20.9%)	12 (16.9%)	27 (19.1%)
Anxiety	9 (29.0%)	16 (39.0%)	0	0	1 (14.3%)	3 (18.8%)	3 (18.8%)	8 (38.1%)	10 (38.5%)	20 (33.9%)
Apathy	28 (56.0%)	33 (51.6%)	12 (60.0%)	16 (76.2%)	5 (25.0%)	19 (47.5%)	19 (54.3%)	18 (41.9%)	36 (52.2%)	97 (67.8%)
Depression	20 (40.8%)	23 (35.9%)	8 (42.1%)	12 (57.1%)	5 (25.0%)	9 (22.5%)	11 (31.4%)	14 (32.6%)	24 (34.8%)	76 (52.8%)
RBD	4 (8.9%)	5 (8.1%)	0	3 (20.0%)	3 (15.8%)	6 (17.1%)	1 (3.0%)	15 (36.6%)	6 (10.2%)	53 (43.8%)

**Motor outcomes**^**a**^										
Bradykinesia	12 (24.0%)	18 (27.7%)	11 (61.1%)	10 (50.0%)	10 (52.6%)	18 (45.0%)	10 (29.4%)	27 (64.3%)	44 (67.7%)	104 (73.2%)
Tremor	5 (10.2%)	8 (12.7%)	4 (22.2%)	6 (28.6%)	3 (15.8%)	6 (15.8%)	6 (17.6%)	14 (35.9%)	18 (27.3%)	62 (44.0%)
Gait change	15 (29.4%)	16 (24.6%)	10 (50.0%)	10 (47.6%)	10 (50.0%)	18 (46.2%)	12 (35.3%)	24 (57.1%)	42 (60.9%)	97 (67.4%)
Falls	11 (21.6%)	4 (6.2%)	5 (26.3%)	5 (23.8%)	2 (10.0%)	9 (25.0%)	3 (9.1%)	15 (34.9%)	21 (30.4%)	49 (34.5%)

**Clinical diagnosis**										
Parkinson's disease	0	2 (3.1%)	2 (9.5%)	0	1 (5.0%)	3 (7.5%)	0	6 (14.0%)	7 (9.9%)	14 (9.7%)
LB dementia	2 (3.9%)	4 (6.2%)	1 (4.8%)	2 (9.5%)	5 (25.0%)	7 (17.5%)	2 (5.7%)	18 (41.9%)	29 (40.8%)	87 (60.0%)
-Parkinson's disease dementia	0	2 (3.1%)	1 (4.8%)	0	1 (5.0%)	3 (7.5%)	0	6 (14.0%)	6 (8.5%)	13 (9.0%)
-Dementia with LB	2 (3.9%)	2 (3.1%)	0	2 (9.5%)	4 (20.0%)	4 (10.0%)	2 (5.7%)	12 (27.9%)	23 (32.4%)	74 (51.0%)
Alzheimer's disease	46 (90.2%)	64 (98.5%)	17 (81.0%)	20 (95.2%)	12 (60.0%)	29 (72.5%)	27 (77.1%)	23 (53.5%)	47 (66.2%)	73 (50.3%)

**ADNC stage**										
-None	1 (2.0%)	0	0	0	2 (10.0%)	3 (7.5%)	2 (5.7%)	4 (9.3%)	0	8 (5.5%)
-Low	0	0	1 (4.8%)	0	4 (20.0%)	9 (22.5%)	1 (2.9%)	9 (20.9%)	4 (5.6%)	23 (15.9%)
-Intermediate	4 (7.8%)	4 (6.2%)	1 (4.8%)	0	7 (35.0%)	13 (32.5%)	4 (11.4%)	8 (18.6%)	21 (29.6%)	43 (29.7%)
-High	46 (90.2%)	61 (93.8%)	19 (90.5%)	21 (100.0%)	7 (35.0%)	15 (37.5%)	28 (80.0%)	22 (51.2%)	46 (64.8%)	71 (49.0%)

Variables are reported as mean (standard deviation) or number (percentage), as appropriate. LB: Lewy body, CDR-SOB: CDR® Dementia Staging Instrument-Sum of Boxes, NPI-Q: Neuropsychiatric Inventory-Questionnaire, RBD: rapid eye movement sleep behavior disorder, ADNC: National Institute on Aging-Alzheimer's Association Alzheimer's disease neuropathologic change. ^**a**^Binary cognitive, behavioral, and motor outcomes are based on the clinician report from the NACC UDS B9 form.

**Table 4:** Clinical features associated with sex within individual LB pathology groups based on models with and without National Institute on Aging-Alzheimer's Association Alzheimer's disease neuropathologic change (ADNC) as a covariate.

	**Sex effect in models without ADNC**	**Sex effect in models with ADNC**
**Amygdala LB**		
Falls	OR (95% CI) = 5.36 (1.42, 26.2), *p =* .020	OR (95% CI) = 5.41 (1.43, 26.5), *p =* .019
**Brainstem LB**		
Agitation	OR (95% CI) = 5.74 (1.20, 32.7), *p =* .034	OR (95% CI) = 7.23 (1.34, 52.4), *p =* .029
Total NPI-Q	B (SE) = 3.19 (1.53), *p =* .042	B (SE) = 3.55 (1.47), *p =* .019
Clinical dementia with LB diagnosis	OR (95% CI) = 6.56 (1.07, 47.9), *p =* .047	OR (95% CI) = 6.68 (1.02, 56.2), *p =* .056
**Limbic LB**		
Memory change	OR (95% CI) = 0.27 (0.031, 1.96), *p =* .21	OR (95% CI) = 0.053 (0.002, 0.72), *p =* .046
Rapid eye movement sleep behavior disorder	OR (95% CI) = 0.060 (0.003, 0.39), *p =* .013	OR (95% CI) = 0.026 (0.0005, 0.34), *p =* .022
Bradykinesia	OR (95% CI) = 0.28 (0.094, 0.81), *p =* .021	OR (95% CI) = 0.32 (0.10, 0.98), *p =* .048
Clinical LB dementia diagnosis	OR (95% CI) = 0.081 (0.011, 0.36), *p =* .003	OR (95% CI) = 0.055 (0.005, 0.34), *p =* .005
Clinical dementia with LB diagnosis	OR (95% CI) = 0.070 (0.007, 0.39), *p =* .007	OR (95% CI) = 0.042 (0.002, 0.34), *p =* .010
**Neocortical LB**		
Language impairment	OR (95% CI) = 2.18 (1.03, 4.90), *p =* .048	OR (95% CI) = 1.62 (0.74, 3.75), *p =* .24
Rapid eye movement sleep behavior disorder	OR (95% CI) = 0.19 (0.069, 0.47), *p <* .001*	OR (95% CI) = 0.25 (0.085, 0.62), *p =* .005*
Clinical LB dementia diagnosis	OR (95% CI) = 0.50 (0.26, 0.94), *p =* .032	OR (95% CI) = 0.65 (0.33, 1.26), *p =* .20

Male sex was the reference group. Findings are reported as odds ratio (95% confidence interval) or B (standard error), as appropriate. Models with cognitive outcomes included age at last visit, informant relationship to participant (spouse/partner or not) and years of education as covariates. Models with clinical diagnosis, behavioral and motor outcomes included age at last visit and informant relationship to participant (spouse/partner or not) as covariates. “With ADNC” column depicts models with ADNC staging as an additional covariate. Rows are shown when at least one model has *p <* .05. Effects with false discovery rate adjusted *q* < 0.05 are marked with *. CDR-SOB: CDR® Dementia Staging Instrument-Sum of Boxes, NPI-Q: Neuropsychiatric Inventory-Questionnaire, OR: odds ratio, CI: confidence interval, SE: standard error.

#### 3.3.1 Amygdala LB

In models with and without ADNC, females had a higher likelihood of falls (*p* < .020 for both), which was not significant after FDR correction (*q* = 0.99 with ADNC as covariate; *q* = 0.61 without ADNC as covariate).

#### 3.3.2 Olfactory LB

There were no significant sex effects in models with and without ADNC before or after FDR correction (*p* > .10 for all).

#### 3.3.3 Brainstem LB

In models without ADNC, females had a higher likelihood of agitation, clinical diagnosis of DLB, and higher NPI-Q scores than males (*p* < .047 for all). In models adjusted for ADNC, sex differences for agitation and NPI-Q scores remained significant (*p* ≤ .029 for both). None of these sex effects were significant after FDR correction (*q* > 0.29 for all).

#### 3.3.4 Limbic LB

In models with and without ADNC, females had a lower likelihood of RBD, bradykinesia, clinical diagnosis of LB dementia including DLB (*p* ≤ .048 for all). In models adjusted for ADNC, females also had a lower likelihood than males to have memory impairment (*p* = .046). None of these sex effects were significant after FDR correction (*q* > 0.056 for all).

#### 3.3.5 Neocortical LB

In models with and without ADNC, females had a lower likelihood of RBD, which remained significant after FDR correction (*q* = 0.022 without ADNC as covariate, *q* = 0.026 with ADNC as covariate). In models without ADNC, females had a higher likelihood of language impairment (*p* = .048), and a lower likelihood of clinical LB dementia diagnosis (*p* = .032), which were not significant in models with ADNC (*p* = .24) or after FDR correction (*q* > 0.28 for both).

## 4 Discussion

This study expands on our previous findings in a cohort of people with limbic and neocortical LB pathology [12], [13] by examining sex differences across the full range of LB pathology stages available in the NACC (Table 5 for summary of the findings). While more advanced LB pathology was associated with a higher likelihood of clinical features characteristic of LB disease, sex modified the associations of LB pathology stage with auditory hallucinations and clinical AD diagnosis. Independent of LB pathology stage and after adjustment for ADNC stage, females were less likely to have RBD and receive a clinical diagnosis of LB dementia. In analyses within individual LB pathology groups, most sex differences did not survive correction for multiple comparisons, except for a lower likelihood of RBD in females with neocortical LB pathology. These findings support sex differences in the clinical expression of LB pathology and suggest that AD co-pathology does not fully account for these differences.

**Table 5:** Summary of clinical outcomes significantly differing by sex after adjustment with ADNC.

	Overall sex differences	Sex interaction with advancing Lewy body pathology
Cognitive outcomes	Limbic LB group: Females had lower likelihood of memory changes	
Behavioral outcomes	Overall cohort: Females had higher likelihood of auditory hallucinations, and lower likelihood of RBD* Brainstem LB group: Females had higher likelihood of agitation and higher Neuropsychiatric Inventory Questionnaire scores Limbic LB group: Females had lower likelihood of RBD Neocortical LB group: Females had lower likelihood of RBD*	Overall cohort: Likelihood of auditory hallucination*, depression, RBD increased more for males
Motor outcomes	Amygdala LB group: Females had higher likelihood of falls Limbic LB: Females had lower likelihood of bradykinesia	Overall cohort: Likelihood of falls increased more for males
Clinical diagnosis	Overall cohort: Females had lower likelihood of LB dementia* and dementia with LB diagnosis Limbic LB group: Females had lower likelihood of LB dementia and dementia with LB diagnosis	Overall cohort: Likelihood of Alzheimer's disease diagnosis* decreased more for males

Statistical significance after false discovery rate correction is marked with *. LB: Lewy body, RBD: rapid eye movement sleep behavior disorder.

Females had more advanced ADNC stages but less advanced LB pathology stages than males. Although the NACC is not designed to estimate population incidence or prevalence, this pattern is consistent with previous sex differences reported in cohorts of community-dwelling older adults [5], [14]. For people with AD pathology, females are more likely to experience an AD clinical profile than males [5]. For people with LB pathology, AD co-pathology is common and associated with a higher likelihood of an AD clinical profile and a lower likelihood of a clinical profile characteristic of LB disease [7], [15]. In our study, more advanced LB pathology was associated with a lower likelihood of clinical AD diagnosis. This association was more pronounced in males, and AD co-pathology did not fully account for the relationship. Female sex was also associated with a lower likelihood of clinical LB dementia diagnosis. The higher prevalence of AD pathology in females [14] and sex differences in the clinical expression of LB pathology may both contribute to differences in how mixed LB and AD pathology presents and is recognized during life. Studies have also reported sex differences in biomarkers and responses to therapeutics in AD and LB diseases [3], [16]. Considering both sex and co-pathology may therefore be important when interpreting clinical and biomarker findings.

Psychosis is more prevalent in clinical LB dementia than in AD during the early stages of dementia [1]. We found that more advanced LB pathology was associated with a greater likelihood of psychotic symptoms, while the association between LB pathology stage and auditory hallucinations differed by sex after adjustment for ADNC. Females showed a higher prevalence of auditory hallucinations at lower LB pathology stages, whereas prevalence increased more markedly with advancing LB pathology stages in males. Compared with males with AD, females with AD have also been reported to have a higher likelihood of experiencing psychosis [17]. The clinical expression of psychosis in people with mixed pathology is likely impacted by both LB and AD pathologies, as well as sex. While visual hallucinations are reported as a more frequent symptom for females than males in some clinically defined cohorts of LB dementia, pathologically defined cohorts have suggested males are more likely to experience visual hallucinations [3]. This discrepancy highlights the importance of pathological confirmation when examining sex differences in the clinical features of LB disease.

RBD showed the most consistent sex difference in our analyses. Higher likelihood of RBD for males than females has been noted in both clinical and community cohorts [18]. RBD is a prodromal feature for LB diseases and is associated with LB pathology and pontomedullary circuits [19]. Consistent with previous studies [20], RBD became more likely with advancing LB pathology stage in our cohort. However, females had a lower likelihood of RBD than males independent of LB pathology stage, and this association persisted after adjustment for ADNC. Moreover, the only sex difference that remained significant after correction for multiple comparisons within individual LB pathology groups was a lower likelihood of RBD in females with neocortical LB pathology, both with and without ADNC adjustment. As neocortical LB is strongly associated with an LB disease clinical profile [7], this sex difference in RBD may be particularly relevant for clinical recognition.

We did not find substantial evidence for sex differences in clinical profile within the amygdala and olfactory LB groups. Amygdala and olfactory LB pathology are generally less strongly associated with an LB disease clinical profile [21]. Amygdala-predominant LB pathology can occur in almost 50% of people with AD [22] and has been associated with worse cognitive decline, higher tau burden and neuronal loss in the hippocampus [23], [24]. Olfactory LB can also occur in people with AD [25], and the amygdala and olfactory LB groups in our cohort had relatively advanced ADNC stages. Olfactory LB can additionally occur in people with an LB disease clinical profile, predict LB pathology in other brain regions, and occur in incidental LB disease, in which pathology is present without parkinsonism or dementia during life [26]. Heterogeneity in accompanying pathology and LB pathology burden may contribute to the different clinical profiles observed at these more restricted LB pathology stages [26]. However, the individual LB groups in our cohort were relatively small, limiting statistical power to detect sex differences.

Our study has several limitations. NACC data are not generalizable to the broader population with LB disease. The majority of the cohort identified as non-Hispanic White and had high levels of education. Access to an AD Research Center may reflect greater access to care and resources. There was an interval of ~2.6 years between the last visit and death, and LB and AD pathology at the time of the final clinical assessment may therefore have differed from that observed at autopsy. Clinical diagnoses in the NACC were based on the diagnostic criteria available at the time of each visit. Other clinical features (e.g., autonomic disturbances, smell loss) are also helpful clinical markers for LB pathology and may differ by sex but were unavailable in the dataset. Some LB pathology groups had relatively small sample sizes, limiting statistical power. LB pathology staging and ADNC reflect an overall distribution and stage of pathology rather than quantitative pathological burden. People within the same pathology stages may therefore differ in pathological burden that could contribute to differences in clinical expression. Informant relationship also differed between females and males in several LB pathology groups, and informant reporting is considered for many of the clinical outcomes included in our analyses. The relative contributions of participant report, informant report, and clinician judgment to the ascertainment of individual clinical features cannot be determined from the NACC data. Although informant relationship was included as a binary covariate in the models, residual differences in symptom recognition or reporting by informants may be particularly relevant for features such as cognitive fluctuations, RBD, and the reported severity and daily impact of cognitive and behavioral symptoms [27], [28].

In conclusion, our findings indicate that the clinical correlates of LB pathology may differ by sex. Females were less likely to have RBD and to receive a clinical diagnosis of LB dementia than males. With advancing LB pathology stage, males were less likely to receive a clinical AD diagnosis than females, and these sex differences were not fully explained by differences in ADNC stage. These findings highlight heterogeneity in clinicopathological relationships among people with LB and AD pathology and support consideration of both sex and co-pathology when interpreting clinical presentation during life.

## Data statement

NACC data are available to qualified researchers by request (naccdata.org).

## CRediT authorship contribution statement

**Ece Bayram:** Writing – original draft, Visualization, Methodology, Funding acquisition, Formal analysis, Conceptualization. **Danelle J. Carter:** Writing – review & editing. **Irene Litvan:** Writing – review & editing, Supervision.

## Ethics declaration

Written informed consent to take part in the study and to publish the article has been obtained from all participants or their legal representatives. The privacy rights of participants have been observed.

This study was performed in compliance with relevant laws, regulatory frameworks and guidelines where the research took place. Ethics committee approval was not required under relevant laws and institutional guidelines. The study was approved by the institutional review board at each participating data collection site and conducted in accordance with the 1964 Declaration of Helsinki. Written informed consent was obtained from participants prior to inclusion.

## Declaration of competing interest

The authors declare the following financial interests/personal relationships which may be considered as potential competing interests: E.B. and D.J.C. declare no competing interests. I.L.’s research is supported by National Institutes of Health (grants U19AG063911, U01NS100610, 2P30AG062429, R01AG085029, U01NS112010, 5U01NS112010/807745, U01NS100610, R25NS098999, U19 AG063911-1, 1R21NS114764-01A1, 2P30 AG062429-06), the Michael J Fox Foundation, the Parkinson’s Foundation, the Lewy Body Association, CurePSP, Roche, AbbVie, Lundbeck, EIP-Pharma, Alterity, Novartis and UCB. She is a member of the Scientific Advisory Board for the Rossy PSP Program at the University of Toronto, Aprinoia and Amydis (gratis). She is Chief Editor of Frontiers in Neurology.
